# The influence of food environments on dietary behaviour and nutrition in Southeast Asia: A systematic scoping review

**DOI:** 10.1177/02601060221112810

**Published:** 2022-07-18

**Authors:** Josephine Gaupholm, Andrew Papadopoulos, Aiza Asif, Warren Dodd, Matthew Little

**Affiliations:** 1 Department of Population Medicine, 3653 University of Guelph, Guelph, ON, Canada; 2 School of Public Health Sciences, University of Waterloo, Waterloo, ON, Canada; 3 School of Public Health and Social Policy, 574711 University of Victoria, Victoria, BC, Canada

**Keywords:** Southeast Asia, food environment, dietary behaviour, nutrition, double burden of malnutrition

## Abstract

**Background:** Food environments are crucial spaces within the food system for understanding and addressing many of the shared drivers of malnutrition. In recent years, food environment research has grown rapidly, however, definitions, measures, and methods remain highly inconsistent, leading to a body of literature that is notably heterogeneous and poorly understood, particularly within regions of the Asia-Pacific. **Aim:** This scoping review aims to synthesize the nature, extent, and range of published literature surrounding the role of the food environment on influencing dietary behaviour and nutrition in Southeast Asia. **Methods:** A systematic search of 5 databases was conducted following PRISMA guidelines for scoping reviews. Eligible studies included peer-reviewed research with adult participants living in Southeast Asia that examined the food environment as a determinant of dietary behaviour or nutrition. **Results:** A total of 45 articles were included. Overall, studies indicated that dietary behaviours in Southeast Asia were primarily driven by social, cultural, and economic factors rather than physical (e.g. geographical) features of food environments. Food price and affordability were most consistently identified as key barriers to achieving healthy diets. **Conclusion:** This work contributes to the establishment of more robust conceptualizations of food environments within diverse settings which may aid future policymakers and researchers identify and address the barriers or obstacles impacting nutrition and food security in their communities. Further research is needed to strengthen this knowledge, particularly research that explicitly explores the macro-level mechanisms and pathways that influence diet and nutrition outcomes.

## Introduction

Malnutrition in all of its forms is the leading cause of poor health globally (Downs et al., 2020; Global Nutrition Report, 2020). The coexistence of both overnutrition (e.g., overweight, obesity, and diet-related non-communicable diseases (NCDs)) and undernutrition (e.g., stunting, wasting, and micronutrient deficiencies) is known as the double burden of malnutrition (DBM) and has consequences well beyond individual health, impacting economic, social, and environmental progress (Afshin et al., 2019). Efforts toward global targets for reducing malnutrition have been – and will continue to be – severely impacted by the COVID-19 pandemic (Dodd et al., 2021; Swinnen and McDermott, 2020). The pandemic has pushed an estimated 155 million people into extreme poverty globally, while the number of individuals suffering from acute hunger was expected to double in 2020 (Dodd et al., 2021; Swinnen and McDermott, 2020). Severe disruptions to global food systems threatens the food security and livelihoods of millions, particularly vulnerable populations such as women, children, older adults, refugees, and the poor (Dodd et al., 2021). In the midst of this crisis, it is imperative to examine the determinants shaping dietary behaviours, not only to combat malnutrition, but to restructure food systems in ways that promote resilience, equity, and sustainability.

Food environments are considered crucial spaces within the food system for understanding and addressing many of the shared drivers of malnutrition (Downs et al., 2020). Defined as the “collective physical, economic, policy, and sociocultural surroundings, opportunities, and conditions that affect people's food and beverage choices and nutritional status”, food environments help us to conceptually understand the complexity of factors that influence diets and nutrition (HLPE, 2017). Existing conceptual frameworks aim to capture both the external factors (i.e., food availability, prices, vendor and product properties, and promotional information) and the internal factors (i.e., food accessibility, affordability, convenience, and desirability) shaping people's food acquisition and consumption practices (Turner et al., 2018). While examination of various types of food environments, such as home, retail, and work food environments, has taken place primarily in high-income country contexts, there has been substantial effort over the last decade to adapt these concepts to drive research in a diversity of other settings (Toure et al., 2021; Turner et al., 2019). This has led to further theoretical advancements including the distinction between natural food environments, which include wild and cultivated food sources, and built food environments, which include formal and informal market food sources (Downs et al., 2020). However, despite these developments, definitions, measures, and methods are not consistently used, resulting in a notably heterogeneous body of literature.

In response to this rapidly growing field of research, efforts have been made to synthesize food environment research from different settings to better capture the state of science within distinct contexts. While several review articles have been recently published exploring food environment research in Latin America and Africa, authors have recognized a notable gap in knowledge synthesis in regions of the Asia-Pacific (Gissing et al., 2017; Osei-Kwasi et al., 2020; Pérez-Ferrer et al., 2019). Indeed, although a systematic scoping review of food environment research from low- and middle-income countries (LMICs) did identify numerous studies from Asian-Pacific countries, these were predominantly from India and China (Turner et al., 2019).

In Southeast Asia, some progress has been made towards achieving global nutrition targets, however, recent data suggest that the prevalence of undernourishment has been stagnant since 2015 while the prevalence of overweight and obesity have been steadily increasing (FAO et al., 2021). However, since 2017 there has been no attempt to systematically consolidate food environment literature in the Asian context and there has never been a review that focused specifically on countries of Southeast Asia. This scoping review aims to bridge this gap by synthesizing the nature, extent, and range of published literature surrounding the role of the food environment on influencing dietary behaviour and nutrition in Southeast Asia.

## Methods

Scoping reviews are particularly useful for synthesizing diverse and emergent bodies of evidence which are harder to capture with more structured review formats such as a systematic review (Grant and Booth, 2009). Food environment research is inherently multidisciplinary and notably heterogeneous, making a scoping review a well-suited approach for mapping this broad range of research. This review was guided by the methodological framework by Arksey and O’Malley (2005) and the subsequent work that has established reporting guidelines and refined the methodology (Levac et al., 2010; Peters et al., 2015; Tricco et al., 2018). Our methods were conducted in accordance with the standards outlined by the Preferred Reporting Items for Systematic reviews and Meta-Analyses (PRISMA) extension for Scoping Reviews checklist (Tricco et al., 2018).

### Conceptual framework

We use the conceptual food environment framework developed by Turner and colleagues (2018) to guide our search strategy and data analysis. They define the food environment as “the interface that mediates people's food acquisition and consumption within the wider food system” and characterize two key domains of food environments (Turner et al., 2018). The external domain consists of extrinsic dimensions such as food availability, prices, vendor and product properties, and marketing and regulation, while the internal (or personal) domain consists of dimensions relating to individuals, including food accessibility, affordability, convenience, and desirability (Turner et al., 2018). Definitions for each dimension are provided in Table 1.

**Table 1.** Definitions of each of the food environment dimensions, as defined by Turner et al. (2018), used in this review.

**External Domain**	*Availability:* the presence or absence of a vendor or product within a given context
	*Prices:* the monetary cost of products
	*Vendors and product properties:* the structural characteristics of vendors (i.e. general type, hours of operation, etc.) and products (i.e. quality, composition, safety, processing, shelf-life, packaging, etc.)
	*Marketing and regulation:* the promotion, advertisement, branding, sponsorship, labelling, and policy regulations relating to the sale of food
**Internal Domain**	*Accessibility:* the collective determinants that dictate an individual's activity as it relates to food acquisition, i.e., distance, time, mobility, modes of transport, etc.
	*Affordability:* an individual's purchasing power
	*Convenience:* the relative time and effort required to obtain, prepare, cook, and consume a food product
	*Desirability:* an individual's preferences, acceptability, tastes, desires, attitudes, culture, knowledge, and skills

### Search strategy

We conducted a systematic search using PubMed, PsycINFO, CABI Global Health, Web of Science Core Collection, and CINAHL. The search included all relevant articles published in English up to December 31, 2020. A research librarian was consulted during the protocol phase of this review and informed the selection of databases and search terms. The search string combined groups of keywords for three main components: geographic location; food environment dimensions; and dietary behaviours/nutrition. Table 2 provides an overview of search terms used and a complete search strategy for Web of Science is presented in the supplementary material. A hand search of the reference lists of included articles was also conducted to ensure all eligible papers were identified.

**Table 2.** Example search terms used in database searches to identify studies on the role of the food environment on influencing dietary behaviour and nutrition in Southeast Asia (* indicates a truncation boolean operator).

***Location Terms***	Southeast* Asia* OR South east* Asia* OR Indonesia* OR Malaysia OR Malay OR Philippines OR Filipino OR Timor-Leste OR East Timor OR Cambodia* OR Laos OR Laotian OR Myanmar OR Burma OR Burmese OR Thai* OR Vietnam* OR Singapore* OR Brunei OR Bruneian
***Food Environment Terms***	food environment* OR nutrition environment* OR nutritional environment* OR eating environment OR foodscape* OR food desert* OR food swamp* OR obesogenic environment* OR nutrition polic* OR food (accept* OR access* OR acqui* OR ad OR ads OR advertis* OR aesthetic* OR afford* OR attitude* OR availab* OR brand* OR composition OR convenience OR cost* OR cultur* OR desir* OR knowledge* OR label* OR marketing OR outlet* OR packag* OR perception* OR polic* OR practice* OR preference* OR prepar* OR price* OR pricing* OR process* OR promot* OR provision* OR purchas* OR quality OR retail OR sale* OR selection OR service* OR shop* OR sponsorship* OR stall* OR store* OR suppl* OR tast* OR vendor*)
***Diet & Nutrition Terms***	diet* OR nutrition OR nutri* intake OR nutri* status OR eating OR food habit* OR food intake* OR food choice* OR food consumption OR energy intake* OR malnutrition OR undernutrition OR underweight OR thinness OR micronutrient deficienc* OR obes* OR overweight

### Study selection

#### Eligibility criteria

Published, peer-reviewed research of any design (including qualitative studies, cohort studies, cross-sectional studies, intervention trials, and systematic reviews) were included if they examined at least one dimension of the food environment (e.g. food availability) as a determinant of dietary behaviour or nutrition among adult (≥ 18 years) populations living in Southeast Asia. Southeast Asia refers to the following 11 countries: Indonesia, Malaysia, Philippines, Timor-Leste (East Timor), Cambodia, Laos, Myanmar (Burma), Thailand, Vietnam, Singapore, and Brunei. Food environment dimensions were defined based on the Turner et al. (2018) framework discussed above. Inclusion was limited to literature available online in English. Book chapters, conference proceedings/abstracts, and news articles were excluded. Studies were excluded if they: only evaluated food fortification or supplementation strategies; studied parental dietary behaviour but only measured child health or nutrition outcomes; and/or included only Southeast Asian migrants in non-Southeast Asian countries.

#### Screening process

All citations were downloaded to DistillerSR (Evidence Partners) for eligibility screening and duplicate detection. Following deduplication, all articles underwent title and abstract (level one) screening by two independent reviewers (J.G. and A.A.) using an eligibility form developed for this review. Articles that passed level one screening proceeded to full text (level two) screening using a more detailed version of the eligibility form, again by the two independent reviewers (J.G. and A.A.). The reviewers met regularly throughout the screening process to resolve conflicts. The Kappa statistics for level one and level two screening were 0.72 (moderate agreement) and 0.81 (strong agreement), respectively (McHugh, 2012).

### Data extraction and synthesis

Data were extracted by the primary reviewer (J.G.) in DistillerSR using a charting form designed for this project. Extracted study characteristics included the year of publication, study location by country and community (if applicable), study design, food environment dimensions assessed, outcome variable(s) description, and primary findings reported. Data were imported into Microsoft Excel for analysis and quality assessment. The latest version of the Mixed Methods Appraisal Tool (MMAT) was used for quality assessment (Hong et al., 2019). Results from the MMAT are presented in the supplementary material.

Due to the multiple methodologies employed by studies, full-text articles were imported into NVivo 12 for thematic analysis (Braun and Clarke, 2006). The usefulness of thematic analysis in scoping reviews has been recently recognized (Levac et al., 2010). Using an iterative approach, we used the food environment framework developed by Turner et al. (2018) as a deductive framework for coding according to the eight dimensions of the food environment. In subsequent steps, codes were refined and sub-themes were identified.

## Results

### General study characteristics

**Figure 1.**
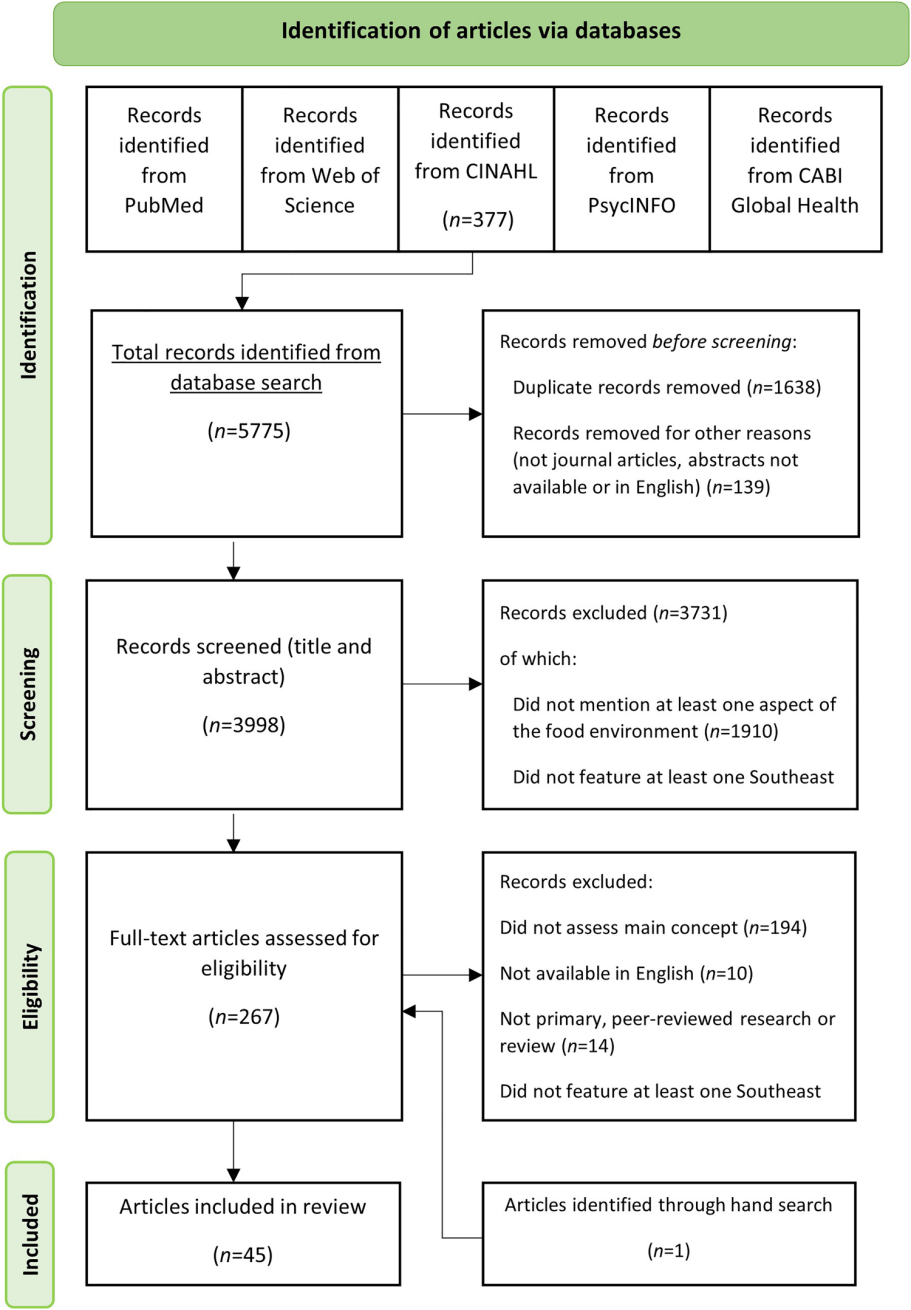
PRISMA flow diagram depicting the four stages of the article selection process for identifying articles on the role of food environments on influencing dietary behaviour and nutrition in Southeast Asia.

**Figure 2.**
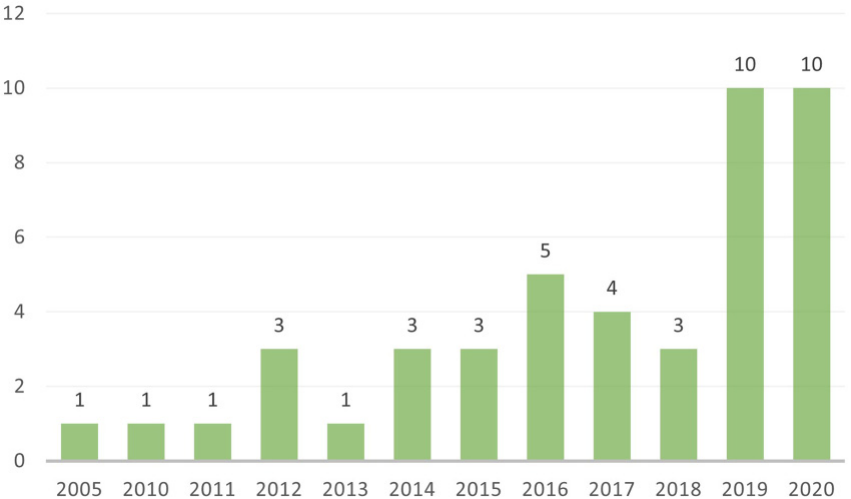
Number of included articles examining the influence of food environments in relation to adult dietary behaviour or nutrition in Southeast Asia per year of publication.

The electronic database search yielded 3998 records after duplicates were removed, of which 267 were eligible for full-text screening (Figure 1). One additional record was identified through our hand search. A total of 45 articles from 39 different studies met the eligibility criteria and were included in this review. Articles are presented by year of publication in Figure 2. The majority (96%) of included articles were published in the last decade, with a noticeable increase in the number of publications in 2019 and 2020. Table 3 provides an overview of the general study characteristics for each article.

**Table 3.** Characteristics of the 45 articles included in this review examining the role of the food environment on dietary behaviour and nutrition in Southeast Asia. (Acc.: Accessibility, Ava.: Availability, Aff.: Affordability, Con.: Convenience, CS: Cross-sectional study, CVD: Cardiovascular disease, Des.: Desirability, EDNP: Energy dense nutrient poor, FFQ: Food Frequency Questionnaire, FGD: Focus group discussion, GIS: Geographic information system, M&R.: Marketing and Regulation, N/A: Not applicable, NHK: Nutritional health knowledge N/R: Not reported, Pri.: Prices, SES: Socioeconomic status, SSCB: Sugar-sweetened carbonated beverages, VPP.: Vendor and Product Properties).

Author(s) (year)	Study Location(s)	Study Design; Methods	Study Setting	Sample Size	Sex	Food Environment Dimension(s) Assessed	Outcome Measure(s)	Summary of Findings
**Abdul Rahman et al. (2013)**	Malaysia	CS; Self-administered food choice questionnaire	Urban	57	Mixed	Ava., Pri., Acc., Aff., Con., Des.	Motives of food choice using point scale	Food choice was primarily motivated by religion, risk perception, & sensory appeal. Food motives were affected by age & income.
**Aji et al. (2019)**	Indonesia	CS; Socio-demographic survey & semi-quantitative FFQ	Costal & mountain community	203	Female	Ava.	Maternal vitamin D & calcium food intake	Place of residence was significantly associated with maternal vitamin D intake but not calcium intake - coastal residents had higher average vitamin D intake.
**Anggraini et al. (2016)**	Indonesia	CS; Survey & semi-quantitative FFQ	Urban poor	188	Female	Ava., VPP., Acc., Aff., Des.	Nutritional status & food consumption	EDNP food consumption was significantly associated with shopping at small shops & street food vendors.
**Asma et al. (2010)**	Malaysia	CS; Interviewer-administered questionnaire	Urban	150 married couples	Mixed	Pri., Con., Des.	Motives of food choice using point scale	Religion, health, & convenience factors were rated as the top motives influencing food choice. Rankings were impacted by SES.
**Baker & Friel, (2016)**	Philippines, Vietnam, Indonesia, Malaysia, Thailand, Singapore, China, Australia, Japan, New Zealand, India, South Korea	Descriptive quantitative study; Analysis of national market share data	National	N/A	N/A	Ava., Pri., M&R.	Trends in ultra-processed food consumption	TFBC market shares & concentration were highest in the carbonated soft drink category. While market concentration in the grocery retail sector is increasing, it still remains low in many Southeast Asian countries.
**Banwell et al. (2016)**	Thailand	Mixed methods; Face-to-face interviews, ethnography, in-depth interviews, & surveys	Urban & rural	1516 (survey) ∼87 (interview, total not reported)	Mixed	Ava., Pri., VPP., M&R., Acc., Aff., Con., Des.	Food purchasing behaviour	Produce was primarily purchased from fresh markets. Preference for purchasing produce from fresh markets driven more by personal, social, & cultural factors.
**Bhanbhro et al. (2020)**	Indonesia	Qualitative; Semi-structured interviews	Rural, Indigenous community	19	Female	Ava., Acc., Aff., Des.	Food consumption patterns during pregnancy	Maternal nutrition & health were facilitated by matrilineal culture & supportive community. Barriers to achieving adequate maternal nutrition & health included poverty, food access, dietary taboos, & inadequate nutritional information.
**Bin Tan & Arcaya (2020)**	Singapore	CS; Surveys & GIS	Urban	529	Mixed	Ava., VPP., Acc.	Self-reported travel time & GIS calculated travel distance	Low-income individuals took a significantly longer time to travel to meal destinations than high-income individuals, largely because they used slower modes of transportation like walking.
**Burger Chakraborty et al. (2016)**	Philippines	Mixed methods; In-depth interviews & national survey data analysis	Urban middle class	30 households	Mixed	Ava., Pri., Acc., Aff., Con., Des.	Food consumption patterns	Processed food consumption driven mainly by convenience & cost-effectiveness. Eating out was more common among younger people & smaller households.
**Charoenbut et al. (2018)**	Thailand	CS; Self-administered questionnaires & interviews	Workplace	26 managers 924 workers	Mixed	Ava., M&R., Acc., Des.	Eating practices of workers & attitude towards healthy eating	No workplace-level factors predicted employee eating practices. At the individual level, attitude towards diet & health was associated with eating practices.
**Chong et al. (2019)**	Malaysia	CS; Face-to-face interview & questionnaires	Rural, Indigenous community	222	Female	Ava., Acc., Aff., Des.	Dietary recall to determine food intake & calculate diet quality score	Household income & nutrition knowledge were positively correlated with diet quality. Predictors of diet quality were marital status, household income, & food security status.
**Colozza (2020)**	Indonesia	Qualitative; Ethnography & in-depth interviews	Urban	45	Mixed	Ava., VPP., Acc., Aff., Des.	Household dietary diversity scores & food acquisition patterns	For both urban & rural residents’, preference for traditional diets remains dominant. Excluding Jakarta, trends in food acquisitions were similar across urban & rural areas, with rural areas showing faster pace of change in some foods.
**Colozza & Avendano (2019)**	Indonesia	Longitudinal study; Secondary data analysis of Indonesia Family Life Survey	Urban & rural	8486	Mixed	Ava., Acc., Des.	Understandings of dietary health, perceptions of healthy food access, & nutritional knowledge	Healthy food was considered highly available. The greatest barrier to healthy eating was food price (specifically animal source products).
**Downs et al. (2019)**	Myanmar	Mixed methods (exploratory sequential); FGD, market & consumer surveys	Urban & rural	32 (FGD) 20 (market survey) 362 (consumer survey)	Female (FGD) Mixed (survey)	Ava., Pri., VPP., M&R., Acc., Aff.	Food preferences, consumption patterns, health & nutrition outcomes	Food availability has increased over time, while food quality is perceived to have declined. Intakes & preferences for processed food remain low. Physical proximity was not seen as a barrier to accessing most food, but affordability was. Children's preferences have a large influence on women's food purchasing.
**Ferzacca et al. (2013)**	Singapore	Qualitative; FGD	Urban	130	Female	Ava., Pri., Acc., Aff., Con., Des.	Food choices & behaviour	Women's food decisions were strongly influenced by their families & inter-generational differences. Women often deferred to their children's preferences when making food decisions.
**Fournier et al. (2016)**	Malaysia	CS; Interviewer-administered survey	National	2000	Mixed	Ava., Acc., Des.	BMI	BMI was significantly associated with; the size of living area, ethnicity, level of education, gender, & age.
**Gan et al. (2020)**	Malaysia	Qualitative; FGD	Rural, Indigenous community	28	Mixed	Ava., VPP., Acc., Aff.	Food seeking behaviour & habits	Barriers to food acquisition included low purchasing power, high food demands, high transport costs, & depletion of wild food sources.
**Goh et al. (2020)**	Malaysia	Quasi-historical quantitative study; Secondary data analysis of national health & nutrition surveys, & food supply data	National	N/R	Mixed	Ava., VPP., M&R., Acc., Con., Des.	Food consumption patterns & prevalence of selected NCDs & risk factors	Nutrition transition linked to; shifting food environment (increased food access & availability); changes in lifestyle & behaviour (increased urbanization, growth of retail culture, exposure to mass media); & government policies (behavioural interventions, agricultural subsidies for NNDFs).
**Harris et al. (2020)**	Vietnam	Quasi-historical quantitative study; Secondary data analysis of national health & nutrition surveys, & food supply data	National	N/R	Mixed	Ava., Pri., M&R.	Changes in diets & nutrition outcomes	Food system drivers of the nutrition transition include economic growth, urbanization, changing food supply & expenditures. However, in Vietnam traditional markets & purchasing activities continue to dominate.
**Hartini et al. (2005)**	Indonesia	Mixed methods; Interviewer-administered questionnaire, FGD, in-depth interviews, & non-participant observations	Urban and rural	450	Female	Pri., Acc., Aff., Des.	Food habits of pregnant women during the economic crisis	Pregnant women reported using simpler cooking methods, buying less food, & focusing more on food taste rather than nutrition. Women did not decrease rice consumption despite the price increase.
**Karupaiah et al. (2013)**	Malaysia	CS/case study; Surveys	Urban women	128	Female	Acc., Aff., Des.	Healthy eating index score	Diet quality was significantly impacted by ethnicity, income, & frequency of eating out. BMI, age, education, & employment status of the women did not appear to be associated with diet quality scores.
**Kelly et al. (2015)**	Thailand	Mixed methods (explanatory sequential); Surveys, & in-depth interviews	National	1516 (survey) 16 (interviews)	Mixed	Ava., Pri., VPP., M&R., Acc., Aff., Con., Des.	Food purchasing & consumption behaviour	Higher-income households had greater access to both supermarkets & fresh markets, as they lived in better-resourced areas. Urban residents were more likely to be frequent supermarket shoppers.
**Kelly et al. (2014)**	Thailand	Cohort; Survey	National	1516	Mixed	Ava., Acc., Aff.	Food shopping patterns	Over half of participants were predominantly fresh market shoppers. Higher-income & urban populations were more likely to buy all food types at modern retailers. Continued use of fresh markets linked to their cultural & social importance. Supermarket use was motivated by atmosphere, car parking, & hygiene.
**Lim et al. (2017)**	Singapore	Secondary data analysis of Singapore Heart Foundation survey data & GIS	Urban poor	1972	Mixed	Acc.	12 CVD risk factors	Increased distance to public amenities was not found to be associated with the majority of CVD risk factors. Only 2 significant associations were found out of 60 tested.
**Lim et al. (2020)**	Singapore	Mixed methods (explanatory sequential); Self-administered survey & in-depth interviews	Urban prediabetic residents	433 (survey) 48 (interview)	Mixed	Ava., VPP., Acc., Aff., Des.	Meeting dietary recommendations	Themes for not meeting dietary recommendations included family influence, taste preferences, lack of food preparation skills, difficulty finding healthy options, & food price.
**Lipoeto et al. (2013)**	Indonesia, Malaysia, Philippines	Mixed methods; FGD, interviews, review of government reports & national surveys	Urban & rural	374 (interview) ∼95 (9 FGD)	Mixed	Ava., Aff., Des.	Food patterns & traditional food consumption	Western & franchise fast foods were consumed minimally. Urban living was significantly associated with consuming more varieties of traditional foods due to increased availability & purchasing power.
**Moxley et al. (2011)**	Philippines	CS; Interviewer-administered survey	Urban & rural	361	Mixed	Acc., Aff., Des.	Nutrition & health knowledge index score	Predictors of NHK were family solidarity, community centrality, information accessibility, & income.
**Naidoo et al. (2017)**	Singapore	Mixed methods (concurrent triangulation); Survey & FGD	Urban	130 (FGD) 1647 (survey)	Female (FGD) Mixed (survey)	Ava., VPP., Aff., Con., Des.	Frequency of eating out	The primary driver of eating out, particularly at hawker centres, was the availability of a large variety of affordable & appealing foods.
**Neo & Brownlee (2017)**	Singapore	Qualitative; Pre & post-FGD	Urban, young adults	30	Mixed	Ava., Pri., VPP., Aff., Con., Des.	Whole-grain food consumption & attitudes	Barriers to wholegrain consumption included personal factors (habits, sensory issues, inability to identify products), product-specific factors (variety, price, convenience) & external factors (family/peer preferences & knowledge, limited availability).
**Ng et al. (2015)**	Malaysia, UK	Qualitative; Camera-based image generation & retrospective in-depth interviews	Urban	18 (10 from Malaysia)	Mixed	Ava., Pri., VPP., Acc., Aff., Des.	Food consumption activities (i.e. planning, procurement, preparation, eating, & disposal)	Price, availability, & food quality influenced where people procured food. Much of what people choose to eat was dictated by routine & sensory appeal, while a lack of knowledge may be a barrier to diet diversity.
**Ong & Kim (2017)**	Philippines	Mixed methods; Semi-structured interviews & WEP identification	Rural, Indigenous community	44	Female	Ava., Acc., Aff., Des.	Wild edible plant use & collection	WEP knowledge & selection were influenced by household income, culture, & past experiences of food scarcity.
**Pawera et al. (2020)**	Indonesia	Mixed methods; Semi-structured interviews, plant identification, & FGD	Rural, Indigenous community	200 (interview) 68 (4 FGD)	Female	Ava., Pri., Acc., Des.	Wild food plant consumption practices	Barriers to WFP consumption were low availability, time constraints, & limited knowledge. Declining WFP consumption linked to decreased availability & changing lifestyle. Strong cultural ties appeared to slow dietary changes.
**Pei et al. (2018)**	Malaysia	CS; Face-to-face interviews	Rural, Indigenous community	222	Female	Ava., Acc.	Diet quality & weight status	Significant associations were found between food insecurity & mean household income, food expenditure, number of children, & household size.
**Rammohan et al. (2019)**	Myanmar	CS; Face-to-face interviews	Rural	3230 households	Mixed	Ava., Acc.	Diet diversity & household food security	Home garden access was positively associated with measures of food security & diet diversity, particularly among landless households. Diet diversity was positively associated with wealth.
**Reyes-García et al. (2019)**	Indonesia	Mixed methods; Semi-structured key-informant interviews, surveys, & FGD	Rural, Indigenous community	393 (160 Baka, 109 Punan Tubu, 124 Tsimane’)	Mixed	Ava., Acc.	Diet diversity score & seasonal dietary patterns	Seasonal variation in diets was reflected in lower diet diversity scores during the rainy season. Diet diversity among the Punan Tubu did not differ significantly between villages with more or less market access.
**Sang-ngoen et al. (2019)**	Thailand	CS; Interviewer-administered questionnaire	Urban and rural	128	Female	Ava., VPP., Acc., Aff., Des.	Food consumption habits	Consumption of ready-to-eat food was positively associated with higher levels of education & income. Significantly more urban women regularly consumed ready-to-eat food. Both urban & Hill tribe women mainly shopped at traditional & mobile markets.
**Sang-ngoen et al. (2020)**	Thailand	CS; Interviewer-administered questionnaire, market survey	Urban and rural	128	Female	Ava., Aff.	Iron & Vitamin C intake	Hill tribe women consumed less iron, animal protein, vitamin C, & calcium than urban women despite similar market availability of iron & vitamin C-rich foods.
**Schram et al. (2015)**	Vietnam, Philippines	Natural experiment; Comparison of SSCB beverage sales pre & post-trade liberalization	National	N/A	N/A	Ava., M&R.	Sales of sugar-sweetened carbonated beverages	Market liberalization in Vietnam led to a significant increase in SSCB sales that was not seen in the control country (Philippines) or in other food sectors that were not expected to be affected. Main beneficiaries of growth were foreign beverage companies.
**Sufyan et al. (2019)**	Indonesia	Qualitative; Semi-structured in-depth interviews	Urban poor	18	Female	Ava., Pri., M&R., Acc., Aff., Con.	Food purchasing behaviour & self-identified BMI	Women's food purchasing behaviour was influenced by time & cost efficiency, food availability, family, & food store marketing strategies.
**Supannee (2020)**	Thailand	CS; Interviewer administered survey	Urban & rural elderly	1336	Mixed	Ava., VPP., Acc., Con., Des.	Food consumption behaviours	Food consumption behaviours were significantly associated with sex, education level, living conditions, knowledge, attitude, & food preferences.
**Trinh et al. (2020)**	Vietnam	Time-series modelling study; Analysis of Vietnam Household Living Standard Survey data & public market data	National	N/R	Mixed	Ava., Acc.	Diet diversity & food basket quality	Wealthier households bought more food & had higher diet diversity scores. The number of supermarkets in the area was associated with per capita expenditure, patterns of food acquisition, & proportion of food consumed.
**Wallace et al. (2014)**	Cambodia	Mixed methods; FGD, interviews, surveys, free-listing, market surveys, & 24-h food recall with pile sort activity	Rural	67	Female	Ava., Pri., Aff., Des.	Iron & vitamin A intakes	Women strongly expressed that poor nutrition was caused by a lack of money & that food insecurity was a daily concern. Food cost & health knowledge were linked to nutritional practice.
**Wertheim-Heck & Raneri (2019)**	Vietnam	Mixed methods (concurrent triangulation); Observation, surveys, in-depth interviews, market census	Urban	31 (retail interview) 6 (street market case studies) 21 (consumer logbooks) 1404 (consumer survey) 183 (retail observation)	Mixed	Ava., Pri., VPP., Acc., Con., Des.	Food shopping practices & diet quality	Geographic proximity to food outlets was not statistically associated with diet quality. The main drivers of continued use of traditional markets included the freshness of products, convenient locations, overall enjoyment, food availability, lower food price, & trusted food safety. All women interviewed indicated they strongly align their food choices with the tastes & preferences of their children.
**Wertheim-Heck et al. (2014)**	Vietnam	Multi-method quantitative study; Food retail census, mapping, & audit, & household survey	Urban poor	400 (survey)	Female	Ava., Pri., VPP., M&R., Acc., Aff., Con., Des.	Nutritional quality, diet diversity, & food safety	95% of vegetables were purchased at informal markets. Daily vegetable purchasing was shaped by either space or time constraints.
**Wertheim-Heck et al. (2019)**	Vietnam	Mixed methods (balanced sequential); Food retail census & audit, household practices & nutrition surveys, multi-generation household interviews, film essay, & stakeholder validation workshop	Urban poor	563 (census) 400 (household practice survey) 347 (nutrition survey) 14 (shopping trips) 14 (interviews) 3 (film essay)	Mixed	Ava., Pri., VPP., M&R., Acc., Aff., Con., Des.	Food consumption practices	Proximity to supermarkets did not influence shopping habits of low-income consumers. Shopping at traditional markets was motivated by habit, availability & variety of foods, convenient location, enjoyment, price, & trust in food safety & freshness.

### Where have studies taken place and what populations have been studied?

**Figure 3.**
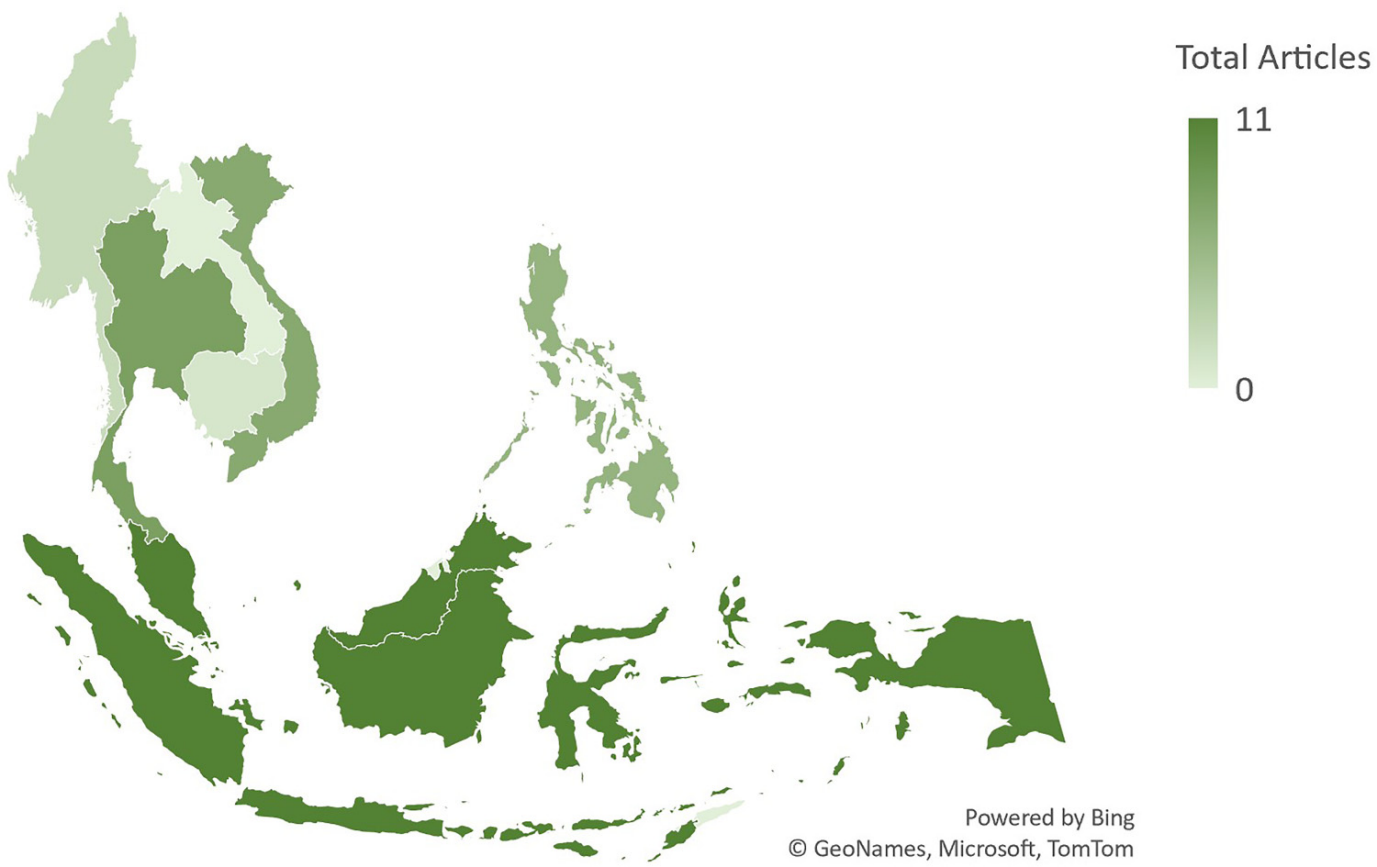
Map of the geographic distribution of the articles included in this review (n = 45). Countries outside of Southeast Asia not depicted.

Figure 3 depicts the geographic distribution of included articles across Southeast Asia. The included articles covered eight of the eleven Southeast Asian countries, with no articles conducted in Laos, Brunei, or Timor-Leste. Indonesia and Malaysia were featured the most with eleven publications each. The majority (97%) of articles were conducted in a single country; however, four studies included multiple locations, two of which featured countries outside of Southeast Asia.

One-third (n = 15) of the articles included only women participants, with an additional two mixed-methods studies having research components that focused only on women. The remaining 28 articles included both men and women. Urban populations were the most frequently studied, with 16 articles focusing solely on urban environments and nine articles comparing urban and rural communities. Nine articles reported on research that included rural Indigenous communities within four different countries.

### What research methods have been used?

Various study designs and methods were utilized across the included articles, as described in Table 3. Over half (n = 24) of the articles used exclusively quantitative approaches, most of which featured a cross-sectional design (63%). The most common quantitative data collection method was interviewer-led or self-administered surveys/questionnaires (n = 17), followed by market-based measures (n = 6), and spatial measurements (n = 4). Seven quantitative articles used secondary data for their analysis.

Approximately 16% of articles (n = 7) exclusively used qualitative methods. Interviews (n = 5) and focus group discussions (n = 3) were the most prominent data collection tools; however, one paper also incorporated an ethnographic component into its design. Fourteen articles featured mixed-method designs and used similar methodologies to those mentioned above, including interviews (n = 12), market-based measures (n = 6), focus groups (n = 7), surveys (n = 10), and/or spatial measures (n = 4).

### What dimensions of the food environment have been assessed?

**Figure 4.**
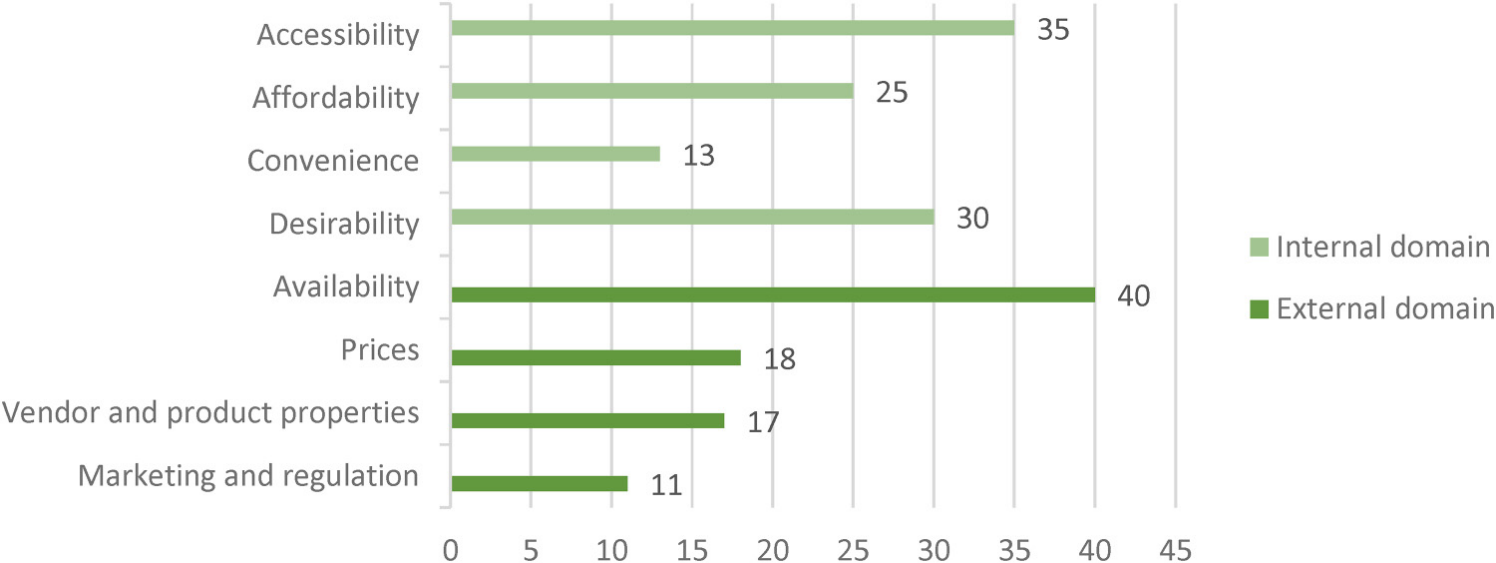
Total number of times each individual food environment dimension was measured in the articles included in this review (n = 45). Numbers are not mutually exclusive, therefore, each article measured a different selection and number of dimensions. .

All food environment dimensions, as defined by Turner et al. (2018), have been studied in Southeast Asia (Figure 4). On average, articles assessed 4 dimensions (standard deviation = 1.9), with four articles measuring all eight dimensions. There were no observable patterns in the combinations of dimensions studied. Of the two central food environment domains (internal and external), the internal domain was most prominent, including articles that assessed accessibility (n = 35), affordability (n = 25), convenience (n = 14), and desirability (n = 30) of food. However, availability was the most studied dimension (n = 40). Thirty-seven of the forty-five publications (82%) addressed both the external and internal food environment.

### How has the food environment been conceptualized?

Eighteen articles directly mentioned the ‘food environment’ as a concept used to frame their research, whereas the other twenty-eight articles only defined the specific food environment dimensions measured but did not explicitly reference the food environment in their work. Of the eighteen papers that used a food environment framework, nearly half (n = 8) focused on the retail food environment, evaluating the geographic distribution and characteristics of various retailing outlets or products in relation to consumer behaviour and/or nutritional outcomes (Anggraini et al., 2016; Bin Tan and Arcaya, 2020; Kelly et al., 2014, 2015; Trinh et al., 2020; Wertheim-Heck et al., 2014, 2019; Wertheim-Heck and Raneri, 2019). Four articles considered the natural food environment and the importance of wild and cultivated plants on diet and nutrition, mainly in the context of subsistence communities (Ong and Kim, 2017; Pawera et al., 2020; Rammohan et al., 2019; Reyes-García et al., 2019). Only one article examined a workplace food environment, assessing both the individual and workplace-level factors influencing eating practices among Thai factory workers (Charoenbut et al., 2018). As this review focused on adult populations, we found no studies that examined school food environments.

### Dietary behaviours and nutrition outcomes

Amongst the included articles, a wide range of outcome variables were examined, with definitions, indicators, and measurement tools not being used uniformly across studies (see Table 3 for specific outcome variables). Of the forty-five articles, thirty (66.7%) reported on outcomes related to food acquisition and consumption patterns, such as food choice motivations, food shopping behaviours or frequency, and food consumption trends. These outcomes were assessed using a range of measurement tools, including surveys, food frequency questionnaires, focus group discussions, in-depth interviews, and observations. Eleven articles assessed dietary intake outcomes, measured primarily as diet diversity and/or diet quality scores using food frequency questionnaires or 24-h food recall data. Nutritional health outcomes were the least studied (n = 5). BMI was measured in all five articles, while the prevalence of various cardiovascular disease risk factors (e.g. hypertension) were reported in two articles. Studies mainly measured these outcomes using anthropometric data or health screening interviews; however, one article used self-reported BMI based on a photographic rating scale.

All five articles looking at nutritional health outcomes focused only on outcomes related to overnutrition (i.e. high BMI) and did not analyse or discuss factors related to undernutrition. Indeed, most articles in this review focused on outcomes of either overnutrition or undernutrition in isolation. The DBM was discussed in only two articles when looking at national trends in underweight and overweight/obesity prevalence. No articles examined DBM outcomes at the community, household, or individual level.

### What are the associations between food environment dimensions and dietary behaviours and nutrition?

#### Availability

Availability was assessed in all but 5 of the included articles. Multiple sources (n = 6) remarked on the steady annual increase in overall food availability in Southeast Asia (Baker and Friel, 2016; Colozza and Avendano, 2019; Goh et al., 2020; Harris et al., 2020; Schram et al., 2015; Trinh et al., 2020). Three of those articles also detailed the parallel increase in availability of highly processed, non-nutrient dense foods (NNDFs) with the increased prevalence of NCDs and obesity (Baker and Friel, 2016; Goh et al., 2020; Harris et al., 2020). Additionally, the presence of modern food outlets, such as supermarkets and fast-food retailers, has increased (Baker and Friel, 2016; Kelly et al., 2014). Despite this, several articles (n = 5) reported retention of traditional diets and acquisition practices across Southeast Asia, including food self-production and fresh market shopping (Bhanbhro et al., 2020; Colozza and Avendano, 2019; Kelly et al., 2015; Lipoeto et al., 2013; Wertheim-Heck et al., 2014). For example, longitudinal analysis of the Indonesia Family Life Survey found budget allocations for foods associated with the local traditional diet (e.g. fish and vegetables) have remained constant over time for both urban and rural residents (Colozza and Avendano, 2019). Similar trends were observed in the Philippines, Malaysia, and Thailand (Bhanbhro et al., 2020; Kelly et al., 2015; Lipoeto et al., 2013).

Perceived availability of healthy food varied between populations. For example, urban residents in Yogyakarta, Indonesia, reported healthy foods were readily available (Colozza, 2020). Meanwhile, in Myanmar, focus group participants from four study settings expressed that a lack of nutritious food was a barrier to consuming healthier diets (Downs et al., 2019). In Thailand, a study comparing nutrient intakes of urban and Hill tribe women found that despite having similar food availability, Hill tribe women consumed significantly less iron, animal protein, vitamin C, and calcium than their urban counterparts (Sang-ngoen et al., 2020).

#### Price

The impact of food prices on diet and nutrition was studied in 18 articles. Time-series data from Vietnam showed that food prices have substantially increased since 1996, with nutrient-rich, diverse diets becoming increasingly expensive, especially when compared to the price of NNDFs (Harris et al., 2020). Focus group discussions among urban and rural women from multiple studies (n = 3) revealed food cost was a major barrier to consuming healthier foods (Downs et al., 2019; Sufyan et al., 2019; Wallace et al., 2014).

Wertheim-Heck and colleagues observed significant differences in the price of vegetables between traditional markets (wet markets or street markets) and supermarkets in Vietnam, with the latter being 35% more expensive (2019). Similarly, consumer surveys and interviews from Thailand revealed that most participants believed fresh produce was substantially cheaper at fresh markets than at supermarkets (Kelly et al., 2015).

#### Vendor and product properties

Researchers often classified vendor and product properties as either ‘modern’ or ‘traditional’. ‘Modern’ was used to characterize national or transnational retailing structures such as supermarkets, convenience stores, and NNDFs, whereas ‘traditional’ referred to long-established retail structures such as wet or open-air markets, mobile vendors, and locally produced, minimally processed food. Several articles (n = 3) used modern retailers as a proxy for food safety due to having greater regulation, advanced infrastructure, and higher hygiene standards (Banwell et al., 2016; Wertheim-Heck et al., 2014, 2019). Findings from Thailand suggest that Thai consumers perceived modern retailers to be more hygienic, clean, and safe than traditional markets (Banwell et al., 2016; Kelly et al., 2015). Other articles used modern retailers to signal obesogenic food environments due to the increased proportion of NNDFs sold at such outlets. Indeed, several studies (n = 5) established significant associations between the frequency of shopping at modern retailers and increased consumption of nutrient-poor foods such as soft drinks, processed meat, and bakery items (Anggraini et al., 2016; Baker and Friel, 2016; Kelly et al., 2014; Sufyan et al., 2019; Trinh et al., 2020).

Vendor and product properties influenced purchasing patterns by catering to different consumer needs. Consumers primarily used modern retailers to purchase packaged and processed foods, while traditional markets were the primary source of fresh and staple foods (Kelly et al., 2015; Wertheim-Heck et al., 2014, 2019; Wertheim-Heck and Raneri, 2019). Results from multiple papers (n = 5) indicated that traditional markets were the preferred and most frequented vending structures among various populations in the region (Banwell et al., 2016; Kelly et al., 2015; Sang-ngoen et al., 2019; Wertheim-Heck et al., 2019; Wertheim-Heck and Raneri, 2019). However, supermarket shopping was positively associated with income, urban living, and smaller household size (Banwell et al., 2016; Kelly et al., 2014; Sang-ngoen et al., 2019).

#### Marketing and regulation

The least studied food environment dimension was marketing and regulation (n = 11). Six articles examined the influence of national policies on consumer shopping trends and/or nutrition outcomes (Baker and Friel, 2016; Banwell et al., 2016; Goh et al., 2020; Harris et al., 2020; Kelly et al., 2015; Schram et al., 2015). For example, in Vietnam, Schram et al. found that sales of sugar-sweetened carbonated beverages rose significantly following market liberalization when compared to a control case, the Philippines (2015). Their analysis also concluded that these trade and investment liberalization policies lead to market domination by foreign transnational food companies (Schram et al., 2015). Similarly, Baker and Friel (2016) described trends in ultra-processed food sales from 2000 to 2013 in Asia, finding that increasing market concentration and trans-nationalization, particularly in the grocery retail sector, are likely key drivers of Asia's nutrition transition.

Regulations at the vendor level were explored in one mixed-method study in the context of safe vegetable provisioning in Hanoi, Vietnam. Wertheim-Heck and colleagues found that less than 3.8% of vegetables sold at traditional markets provided any sort of food safety claim, such as product labelling or food safety certification, however, despite this, over 95% of total vegetables consumed by the study population were sold at traditional markets (Wertheim-Heck et al., 2019; Wertheim-Heck and Raneri, 2019).

#### Accessibility

Numerous articles (n = 35) explored the variable effects of food accessibility on dietary behaviours and nutrition. Several articles (n = 5) reported non-significant relationships between geographic proximity to food vendors and dietary outcomes, including measures of dietary diversity and food shopping patterns, across a range of populations (Downs et al., 2019; Lim et al., 2017; Trinh et al., 2020; Wertheim-Heck et al., 2014, 2019). However, transportation access was found to influence food acquisition, particularly among rural and low-income populations. For example, qualitative evidence from multiple rural Indigenous communities reported that long travel times and high transportation costs limited participants' ability to acquire food (Bhanbhro et al., 2020; Gan et al., 2020). Similarly, in both Singapore and Thailand, it was observed that low-income populations expended more time acquiring food as they tended to use slower modes of transportation, such as walking or riding bicycles, and often had to travel greater distances than high-income populations (Bin Tan and Arcaya, 2020; Kelly et al., 2014).

Accessibility of natural food environments, including wild and cultivated food sources, was studied in four articles and found to positively impact nutrition and diet (Ong and Kim, 2017; Pawera et al., 2020; Rammohan et al., 2019; Reyes-García et al., 2019). For instance, in rural Myanmar, access to a home garden was associated with greater levels of food security and higher dietary diversity scores (Rammohan et al., 2019).

#### Affordability

Twenty-five articles looked at the impact of affordability on dietary behaviour and nutrition. Several qualitative articles (n = 5) reported affordability and lack of purchasing power were major barriers to achieving healthy and adequate diets (Bhanbhro et al., 2020; Colozza, 2020; Hartini et al., 2005; Neo and Brownlee, 2017; Sufyan et al., 2019). Similarly, quantitative evidence found significant and positive correlations between household income and diet quality (Chong et al., 2019; Karupaiah et al., 2013), food security (Pei et al., 2018), and nutrient intakes (Sang-ngoen et al., 2019, 2020).

Perceived affordability varied by food item. Vegetables were widely regarded as inexpensive, particularly when sold by traditional vendors (Colozza, 2020; Downs et al., 2019; Pawera et al., 2020), whereas fruit, meat, and other animal products were considered expensive by research participants (Colozza, 2020; Sufyan et al., 2019). Women in Singapore and East Jakarta, Indonesia, reported that purchasing ready-made food was more affordable than cooking at home for their families (Naidoo et al., 2017; Sufyan et al., 2019).

#### Convenience

Convenience, related to vendor properties (e.g. opening hours), product properties (e.g. ready-to-eat), and accessibility (e.g. market location), was examined in 13 articles. Women described a lack of time and busy work schedules as reasons for eating out or purchasing ready-to-eat meals in Singapore (Ferzacca et al., 2013; Naidoo et al., 2017) and Indonesia (Sufyan et al., 2019). Household income was correlated with convenience as a motive for food choice in two studies, with lower-income households valuing convenience more highly than those with higher incomes (Abdul Rahman et al., 2013; Asma et al., 2010). Several articles (n = 3) noted that the preferred time to purchase vegetables was in the early morning; consequently, among urban residents in Hanoi, Vietnam, traditional markets were considered to have more convenient opening hours and locations (Wertheim-Heck et al., 2014; Wertheim-Heck and Raneri, 2019).

#### Desirability

Desirability, as defined in the Turner et al. framework (2018), encapsulates several interconnected factors important to diets and nutrition. We discuss the three main components that emerged from our analysis, including, social and cultural factors, food preferences, and knowledge.

#### Social and cultural factors

Social influences were highlighted predominantly within qualitative and mixed-method studies (Anggraini et al., 2016; Bhanbhro et al., 2020; Ferzacca et al., 2013; Lim et al., 2020; Moxley et al., 2011; Neo and Brownlee, 2017; Ng et al., 2015; Sufyan et al., 2019). Living with extended family or in multi-generational homes is common in Asian cultures, and there is research to suggest it contributes to healthier dietary behaviours (Sufyan et al., 2019; Supannee, 2020). A study conducted in northeastern Thailand reported that the odds of elderly adults having good food consumption behaviours, classified by a 4-point rating scale, were 2.2 times higher among those living with family compared to those living alone (Supannee, 2020). Marital status was also found to be positively associated with diet quality (Chong et al., 2019) and meeting dietary recommendations (Lim et al., 2020).

Social networks were also seen to impact dietary practices. Evidence from both Thailand and Vietnam indicated that consumers viewed food shopping as a valuable social activity (Banwell et al., 2016; Kelly et al., 2015; Wertheim-Heck et al., 2014). These studies illustrated the importance of fresh market shopping in study participants' daily routines, noting that the motivation behind the choice of retail site was influenced by consumers’ relationships with specific vendors (Banwell et al., 2016; Wertheim-Heck et al., 2014).

Cultural factors, including religion, traditional foods and customs, and ethnicity, were also important determinants of dietary behaviour. Two articles from Malaysia reported that religion was ranked as the most important factor in food selection, above other factors including price, food safety, and health (Abdul Rahman et al., 2013; Asma et al., 2010). Additionally in Malaysia, ethnicity was associated with BMI (Fournier et al., 2016) and diet quality (Karupaiah et al., 2013).

#### Food preferences

Food preferences were another common driver of dietary behaviours. Women reported family food preferences, particularly children's food preferences, strongly influenced food purchasing decisions (Downs et al., 2019; Ferzacca et al., 2013; Sufyan et al., 2019; Wertheim-Heck and Raneri, 2019). In Singapore, women discussed navigating differences in family food preferences, describing how eating take-out or “outside food” was a common strategy for satisfying intergenerational tastes (Ferzacca et al., 2013). Shifting food preferences were reported (n = 4) between generations. Older generations often expressed concern about the younger generation's preference for NNDFs given the expansion and popularity of western-style foods and brands (Downs et al., 2019; Ferzacca et al., 2013; Naidoo et al., 2017; Pawera et al., 2020). Among two Indigenous communities in West Sumatra, Indonesia decreased consumption of certain traditional wild plants was attributed in part to younger generations disliking the taste (Pawera et al., 2020). However, in numerous other articles (n = 5) traditional diets were found to be preferred (Banwell et al., 2016; Colozza and Avendano, 2019; Kelly et al., 2014; Lipoeto et al., 2013; Wertheim-Heck et al., 2019).

#### Nutrition knowledge

Six articles identified links between nutrition knowledge and dietary outcomes. For example, in Malaysia, cross-sectional evidence reported positive correlations between nutrition knowledge and diet quality (Chong et al., 2019), and level of education and BMI (Fournier et al., 2016). Qualitative evidence from multiple settings revealed that limited nutrition knowledge inhibited people from trying new or unfamiliar foods, such as whole grains and fruit (Neo and Brownlee, 2017; Ng et al., 2015). Additionally, in Indonesia, Indigenous farmers cited a lack of knowledge cultivating and cooking traditional plants as a barrier to consuming them (Pawera et al., 2020).

## Relationship between food environment dimensions

**Figure 5.**
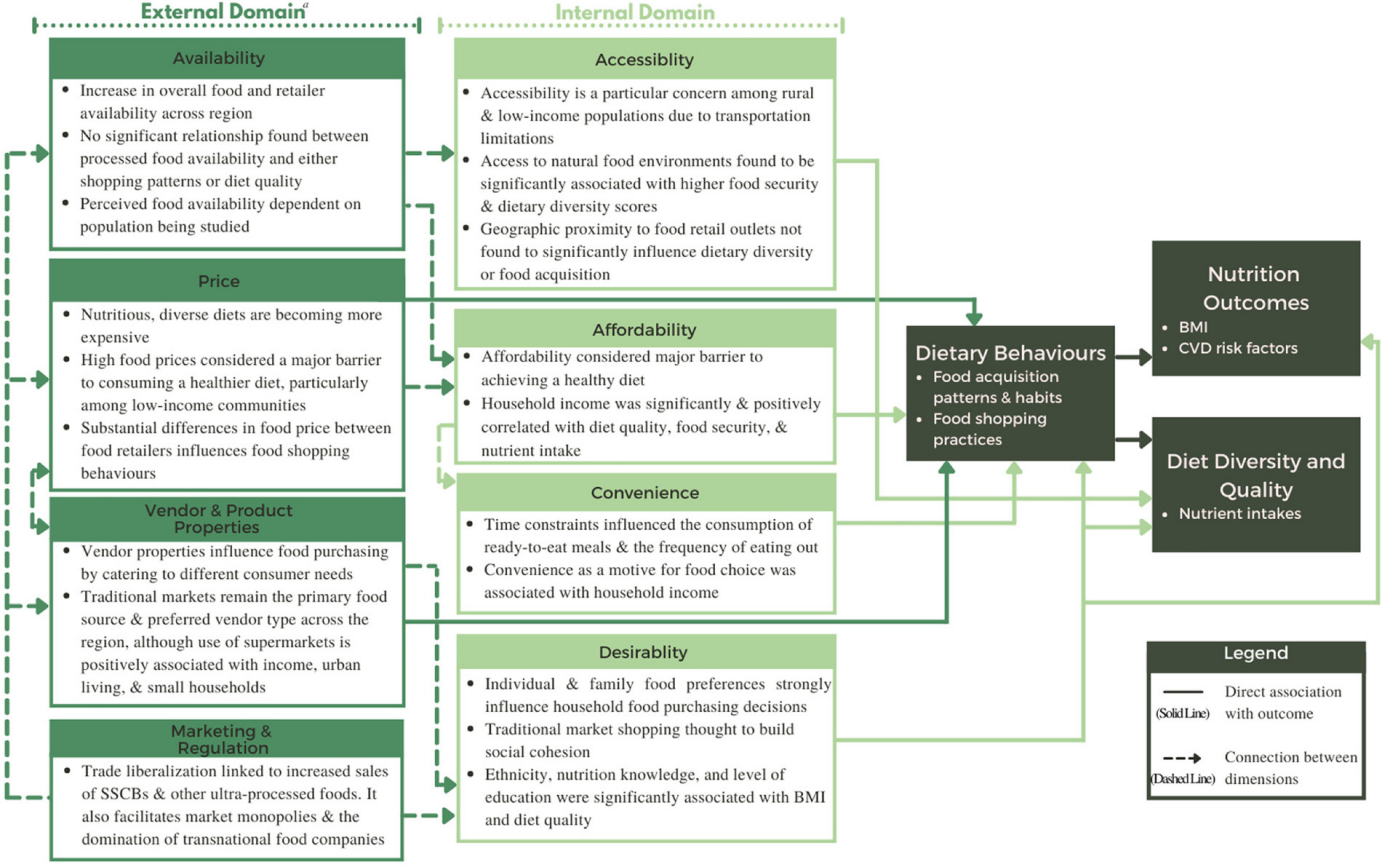
Summary of the relationship between food environment dimensions and dietary behaviour and nutrition outcomes in Southeast Asia based on scoping review (n = 45). ^a^ See supplementary material for list of references. (BMI: Body mass index, SSCB: Sugar-sweetened carbonated beverage)

Each of the eight dimensions of the food environment, while distinct, are intrinsically interconnected. We identified multiple connections between the various dimensions, as depicted in Figure 5. Notably, we saw that the influence of marketing and regulation on diet and nutrition outcomes was observed indirectly through its effect on other food environment dimensions such as availability or price. Indeed, we see multiple instances of the external domain exerting influence on dimensions of the internal domain.

## Discussion

This review aimed to explore and synthesize literature pertaining to the role of the food environment in determining dietary behaviour and nutrition in Southeast Asia. We identified 45 articles from 39 studies that investigated the effect of food environments on dietary behaviour and nutrition in eight Southeast Asian countries. These studies used various study designs and assessment methods to investigate food environment dimensions and outcome measures. Studies most commonly used quantitative design methodologies, followed by mixed-method approaches and then qualitative designs. The majority of articles reported on outcomes related to food acquisition and consumption patterns, while only five articles examined nutritional health outcomes. This is consistent with other reviews of food environment literature which have found an emphasis on quantitative research designs, in addition to a lack of evidence related to nutritional health outcomes (Pérez-Ferrer et al., 2019; Turner et al., 2019).

Overall, we found a paucity of evidence clearly articulating the conceptualization of food environments in this region. Of the studies that explicitly used a food environment framework, most did not define or explain how these concepts were being applied. Further, while urban and retail food environments featured prominently, workplace food environments and natural food environments were given little attention. All eight dimensions of the food environment were assessed by various studies, with the internal domain featuring most prominently. This differs from the results of a scoping review published in 2019, which found the external domain to be the primary focus of food environment research within LMICs (Turner et al., 2019). These opposing results could result from the differences in scope and year of publication between the two reviews (Turner et al., 2019).

Availability and accessibility were the two most frequently assessed dimensions of the food environment amongst included articles but were shown to have variable effects on dietary behaviour and nutrition. Their prominence in the literature may reflect the influence of the food environment discourse taking place in high-income country settings, which often focuses on quantifying characteristics related to food availability and accessibility (Turner et al., 2018). It is well documented that the proliferation of modern food outlets and ultra-processed food supply is driving the increased prevalence of overnutrition and diet-related NCDs globally, known as the nutrition transition (Popkin et al., 2020). However, consistent with other reviews (Caspi et al., 2012; Cobb et al., 2015; Pérez-Ferrer et al., 2019), we found limited evidence statistically linking nutritional health outcomes with increased availability or consumption of processed foods in Southeast Asia (Colozza, 2020; Harris et al., 2020; Kelly et al., 2014; Wertheim-Heck et al., 2019; Wertheim-Heck and Raneri, 2019). While these findings may call for further recognition regarding the importance of other food environment dimensions, such as affordability and desirability, they do not necessarily discount the influence of availability and accessibility. Indeed, such failure to measure the nutrition transition, particularly in LMICs, is strongly linked to inappropriate dietary assessment tools which do not accurately capture shifts in ultra-processed food consumption or foods consumed outside the home (Walls et al., 2018).

Actions targeting food environments as a way to improve diets and nutrition must be informed by the specific needs and characteristics of the target population. Notably, we found considerable research documenting the retention of traditional diets and consumption patterns across the region. Practices related to traditional food cultures, such as shopping at fresh or wet markets, were observed to hold high social value and were a means of cultivating community and cultural solidarity (Banwell et al., 2016; Kelly et al., 2015; Wertheim-Heck et al., 2014). Policymakers can leverage these strong cultural traditions in ways that intentionally cultivate traditional healthy food customs and support sustainable diets and good nutrition. Indeed, actions fostering traditional food cultures, consumption, and practices have been identified as one of thirteen global nutrition initiatives for promoting sustainable healthy diets (Reyes et al., 2021).

Through our analysis, we identified that the influence of social, cultural, and economic factors on dietary behaviours were more strongly emphasized in the literature rather than physical (e.g. geographical) factors, which are common in western contexts. Food price and affordability were regularly considered key barriersto achieving healthy diets. Studies reported significant associations between household income and diet quality (Chong et al., 2019; Karupaiah et al., 2013), food security (Pei et al., 2018), and nutrient intakes (Sang-ngoen et al., 2019, 2020), while qualitative evidence repeatedly indicated that high food cost and low incomes were considered main contributors of poor nutrition (Bhanbhro et al., 2020; Colozza, 2020; Gan et al., 2020; Neo and Brownlee, 2017; Sufyan et al., 2019). This is of considerable concern, particularly given that global food prices are rising in the wake of the COVID-19 pandemic (Swinnen and McDermott, 2020).

We identified several gaps in the literature that highlight areas for additional research and attention. Echoing the sentiments of numerous other reviews (Caspi et al., 2012; Cobb et al., 2015; Gissing et al., 2017; Lytle and Sokol, 2017; Osei-Kwasi et al., 2020; Pérez-Ferrer et al., 2019; Turner et al., 2019), the inconsistency of food environment definitions and methods compromises our ability to learn from and apply research findings adequately. Developing standardized methods for studying food environments in diverse settings would allow for comparisons across regions and lead to better-informed nutrition programmes and policies. In addition, to generate a more comprehensive understanding of the factors influencing dietary behaviours and nutrition in Southeast Asia, it is necessary to expand the scope of research to include measures of both the built food environment and the natural food environment. Finally, effort is required to connect the marketing, regulation, and policy components of the food environment more fully with the other dimensions. We found marketing and regulation to be the least studied dimension of the food environment. While literature emerging from other disciplines (e.g., economics and political science) documents the economic and political dimensions of food systems, there is an absence of research that explicitly explores the macro-level mechanisms and pathways that influence diet and nutrition outcomes (Caspi et al., 2012; Larson and Story, 2009; Osei-Kwasi et al., 2020). This underscores the need for interdisciplinary food environment research in order to improve the integration between the internal and external domains of the food environment.

## Study limitations

This review was subject to several limitations. First, we were restricted to articles available in English which may have excluded relevant research published in other languages. Second, due to the nature of food environment literature, there was substantial heterogeneity within study characteristics and design. The range of study populations, indicators, measurement tools, and study designs limits our ability to integrate and compare research findings. Third, this review does not cover literature published after December 2020 and does not capture any emerging research on the impacts of the COVID-19 pandemic. Due to the profound and ongoing disruptions resulting from the pandemic, food environments, diets, and nutrition outcomes described in this review may not be reflective of the current circumstances in Southeast Asia.

## Conclusion

Findings from this review reaffirm the highly contextual nature of dietary behaviours, which depend on complex interconnected personal and external factors. We found that while all dimensions of the food environment have been studied in Southeast Asia, there is a need to better integrate the external and internal domains to explore specific mechanisms and pathways that influence diet and nutrition outcomes. In recent years, there has been a notable rise in publications examining food environments, signalling growing recognition of their importance and role in mediating diets and nutrition. It is widely recognized that substantial, sustainable progress in combatting malnutrition requires long-term, system-wide change. Establishing a robust conceptualization of the food environment would ensure policymakers, researchers, and programme developers are able to identify and address the barriers or obstacles that impact nutrition and food security in their communities.

## Supplemental Material

sj-docx-1-nah-10.1177_02601060221112810 - Supplemental material for The influence of food environments on dietary behaviour and nutrition in Southeast Asia: A systematic scoping review Supplemental material, sj-docx-1-nah-10.1177_02601060221112810 for The influence of food environments on dietary behaviour and nutrition in Southeast Asia: A systematic scoping review by Josephine Gaupholm, Andrew Papadopoulos, Aiza Asif, Warren Dodd and Matthew Little in Nutrition and Health
