# Supplementary material for: The influence of food environments on dietary behaviour and nutrition in Southeast Asia: A systematic scoping review
Source: Nutr Health. 2022 Jul 18;29(2):231–53. doi: 10.1177/02601060221112810 (PMC10114263; doi:10.1177/02601060221112810)
Supplement: sj-docx-1-nah-10.1177_02601060221112810 - Supplemental material for The influence of food environments on dietary behaviour and nutrition in Southeast Asia: A systematic scoping review [file sj-docx-1-nah-10.1177_02601060221112810.docx]

**Supplementary Material**

*Table of Contents*

Table S1: Preferred Reporting Items for Systematic reviews and Meta-Analyses extension for Scoping Reviews (PRISMA-ScR) Checklist. Adapted from Tricco et al., 2018…………………………………….2

Table S2: Complete search strategy for Web of Science Core Collection. Filtered for English…………...3

Table S3: Quality assessment using the Mixed-Methods Appraisal Tool………………………………….4

Table S4: Reference sources used in Figure 5 of manuscript………………………………………………6

## Table S1: Preferred Reporting Items for Systematic reviews and Meta-Analyses extension for Scoping Reviews (PRISMA-ScR) Checklist. Adapted from Tricco et al., 2018.

| SECTION | ITEM | PRISMA-ScR CHECKLIST ITEM | REPORTED ON PAGE # |
| --- | --- | --- | --- |
| **TITLE** | | | |
| Title | 1 | Identify the report as a scoping review. | 1 |
| **ABSTRACT** | | | |
| Structured summary | 2 | Provide a structured summary that includes (as applicable): background, objectives, eligibility criteria, sources of evidence, charting methods, results, and conclusions that relate to the review questions and objectives. | 1 |
| **INTRODUCTION** | | | |
| Rationale | 3 | Describe the rationale for the review in the context of what is already known. Explain why the review questions/ objectives lend themselves to a scoping review approach. | 2 |
| Objectives | 4 | Provide an explicit statement of the questions and objectives being addressed with reference to their key elements (e.g., population or participants, concepts, and context) or other relevant key elements used to conceptualize the review questions and/or objectives. | 3 |
| **METHODS** | | | |
| Protocol and registration | 5 | Indicate whether a review protocol exists; state if and where it can be accessed (e.g., a Web address); and if available, provide registration information, including the registration number. | 4 |
| Eligibility criteria | 6 | Specify characteristics of the sources of evidence used as eligibility criteria (e.g., years considered, language, and publication status), and provide a rationale. | 5 |
| Information sources | 7 | Describe all information sources in the search (e.g., databases with dates of coverage and contact with authors to identify additional sources), as well as the date the most recent search was executed. | 4 |
| Search | 8 | Present the full electronic search strategy for at least 1 database, including any limits used, such that it could be repeated. | 4 & supp material pg. 4 |
| Selection of sources of evidence | 9 | State the process for selecting sources of evidence (i.e., screening and eligibility) included in the scoping review. | 4 & 5 |
| Data charting process | 10 | Describe the methods of charting data from the included sources of evidence (e.g., calibrated forms or forms that have been tested by the team before their use, and whether data charting was done independently or in duplicate) and any processes for obtaining and confirming data from investigators. | 5 |
| Data items | 11 | List and define all variables for which data were sought and any assumptions and simplifications made. | 5 |
| Critical appraisal of individual sources | 12 | If done, provide a rationale for conducting a critical appraisal of included sources of evidence; describe the methods used and how this information was used in any data synthesis (if appropriate). | 5 |
| Synthesis of results | 13 | Describe the methods of handling and summarizing the data that were charted. | 5 |
| **RESULTS** | | | |
| Selection of sources of evidence | 14 | Give numbers of sources of evidence screened, assessed for eligibility, and included in the review, with reasons for exclusions at each stage, ideally using a flow diagram. | 5 & 6 |
| Characteristics of sources of evidence | 15 | For each source of evidence, present characteristics for which data were charted and provide the citations. | 9-13 |
| Critical appraisal within sources of evidence | 16 | If done, present data on critical appraisal of included sources of evidence (see item 12). | Supp material pg. 5 & 6 |
| Results of individual sources of evidence | 17 | For each included source of evidence, present the relevant data that were charted that relate to the review questions and objectives. | 9-13 |
| Synthesis of results | 18 | Summarize and/or present the charting results as they relate to the review questions and objectives. | 14-21 |
| **DISCUSSION** | | | |
| Summary of evidence | 19 | Summarize the main results (including an overview of concepts, themes, and types of evidence available), link to the review questions and objectives, and consider the relevance to key groups. | 22-24 |
| Limitations | 20 | Discuss the limitations of the scoping review process. | 24-25 |
| Conclusions | 21 | Provide a general interpretation of the results with respect to the review questions and objectives, as well as potential implications and/or next steps. | 25 |
| **FUNDING** | | | |
| Funding | 22 | Describe sources of funding for the included sources of evidence, as well as sources of funding for the scoping review. Describe the role of the funders of the scoping review. | 26 |

## Table S2: Complete search strategy for Web of Science Core Collection. Filtered for English.

| **#** | **Search** | **Results** |
| --- | --- | --- |
| 1 | TS=("Southeast Asia" OR "Southeastern Asia" OR "Southeast Asian" OR "South east Asia" OR "South east Asian" OR "South-east Asia" OR "South-east Asian" OR Indonesia* OR Malaysia OR Malay OR Philippines OR Filipino OR Timor-Leste OR "East Timor" OR Cambodia* OR Laos OR Laotian OR Myanmar OR Burma OR Burmese OR Thai* OR Vietnam* OR Singapore* OR Brunei OR Bruneian) | 350,190 |
| 2 | ALL=("food environment$" OR "nutrition environment$" OR "nutritional environment$" OR "eating environment$" OR foodscape$ OR "food desert$" OR "food swamp$" OR "obesogenic environment$") | 6,385 |
| 3 | TS=(food NEAR/1 (accept* OR access* OR acqui* OR ad OR ads OR advertis* OR aesthetic* OR afford* OR attitude* OR availab* OR brand* OR composition OR convenience OR cost* OR cultur* OR desir* OR knowledge* OR label* OR marketing OR outlet* OR packag* OR perception* OR polic* OR practice* OR preference* OR prepar* OR price* OR pricing* OR process* OR promot* OR provision* OR purchas* OR quality OR retail OR sale* OR selection OR service* OR shop* OR sponsorship* OR stall* OR store* OR suppl* OR tast* OR vendor*)) | 140,957 |
| 4 | #2 OR #3 | 144,768 |
| 5 | ALL=("energy intake$" OR eating OR malnutrition OR undernutrition OR "under nutrition" OR under-nutrition OR underweight OR under-weight OR "under weight" OR thinness OR "micronutrient deficienc*" OR obes* OR overweight OR "over weight" OR over-weight) | 663,838 |
| 6 | TS=(nutri* NEAR/1 (intake$ OR status)) | 63,441 |
| 7 | TS=(food NEAR/1 (habit$ OR intake$ OR choice$ OR consumption)) | 100,718 |
| 8 | ALL=(Diet* OR nutrition ) | 1,621,899 |
| 9 | #5 OR #6 OR #7 OR #8 | 2,098,392 |
| 10 | #1 AND #4 AND #9 | 1,136 |
| 11 | TS=(infant* OR adolescen* OR teen* OR youth* OR juvenile* OR baby OR babies OR girl OR boy OR toddler*) | 1,334,605 |
| 12 | #10 NOT #11 | 982 |

## Table S3: Quality assessment using the Mixed-Methods Appraisal Tool

| Author(s) (year) | 1.1 | 1.2 | 1.3 | 1.4 | 1.5 | 3.1 | 3.2 | 3.3 | 3.4 | 3.5 | 4.1 | 4.2 | 4.3 | 4.4 | 4.5 | 5.1 | 5.2 | 5.3 | 5.4 | 5.5 |
| --- | --- | --- | --- | --- | --- | --- | --- | --- | --- | --- | --- | --- | --- | --- | --- | --- | --- | --- | --- | --- |
| Abdul Rahman et al. (2013) |  |  |  |  |  |  |  |  |  |  | 🗸 | X | C/D | X | 🗸 |  |  |  |  |  |
| Aji et al. (2019) |  |  |  |  |  | X | 🗸 | 🗸 | X | 🗸 |  |  |  |  |  |  |  |  |  |  |
| Anggraini et al. (2016) |  |  |  |  |  | 🗸 | 🗸 | 🗸 | 🗸 | 🗸 |  |  |  |  |  |  |  |  |  |  |
| Asma et al. (2010) |  |  |  |  |  | X | 🗸 | 🗸 | C/D | 🗸 |  |  |  |  |  |  |  |  |  |  |
| Baker & Friel, (2016) |  |  |  |  |  |  |  |  |  |  | 🗸 | 🗸 | 🗸 | C/D | 🗸 |  |  |  |  |  |
| Banwell et al. (2016) | 🗸 | 🗸 | C/D | 🗸 | 🗸 | 🗸 | 🗸 | 🗸 | 🗸 | 🗸 |  |  |  |  |  | 🗸 | X | 🗸 | 🗸 | 🗸 |
| Bhanbhro et al. (2020) | 🗸 | 🗸 | 🗸 | 🗸 | 🗸 |  |  |  |  |  |  |  |  |  |  |  |  |  |  |  |
| Bin Tan & Arcaya, (2020) |  |  |  |  |  | X | 🗸 | 🗸 | 🗸 | 🗸 |  |  |  |  |  |  |  |  |  |  |
| Burger Chakraborty et al. (2016) | 🗸 | 🗸 | C/D | 🗸 | X |  |  |  |  |  | 🗸 | 🗸 | X | C/D | C/D | 🗸 | X | X | C/D | X |
| Charoenbut et al. (2018) |  |  |  |  |  | C/D | 🗸 | 🗸 | 🗸 | 🗸 |  |  |  |  |  |  |  |  |  |  |
| Chong et al. (2019) |  |  |  |  |  | 🗸 | 🗸 | 🗸 | 🗸 | 🗸 |  |  |  |  |  |  |  |  |  |  |
| Colozza, (2020) | 🗸 | 🗸 | 🗸 | 🗸 | 🗸 |  |  |  |  |  |  |  |  |  |  |  |  |  |  |  |
| Colozza & Avendano, (2019) |  |  |  |  |  | C/D | 🗸 | 🗸 | 🗸 | 🗸 |  |  |  |  |  |  |  |  |  |  |
| Downs et al. (2019) | 🗸 | 🗸 | 🗸 | 🗸 | 🗸 |  |  |  |  |  | 🗸 | 🗸 | 🗸 | C/D | 🗸 | 🗸 | 🗸 | 🗸 | 🗸 | 🗸 |
| Ferzacca et al. (2013) | 🗸 | 🗸 | 🗸 | 🗸 | 🗸 |  |  |  |  |  |  |  |  |  |  |  |  |  |  |  |
| Fournier et al. (2016) |  |  |  |  |  | 🗸 | 🗸 | 🗸 | 🗸 | 🗸 |  |  |  |  |  |  |  |  |  |  |
| Gan et al. (2020) | 🗸 | 🗸 | 🗸 | 🗸 | 🗸 |  |  |  |  |  |  |  |  |  |  |  |  |  |  |  |
| Goh et al. (2020) |  |  |  |  |  |  |  |  |  |  | 🗸 | 🗸 | X | C/D | C/D |  |  |  |  |  |
| Harris et al. (2020) |  |  |  |  |  |  |  |  |  |  | 🗸 | C/D | 🗸 | C/D | 🗸 |  |  |  |  |  |
| Hartini et al. (2005) | 🗸 | 🗸 | 🗸 | 🗸 | 🗸 | X | X | 🗸 | C/D | 🗸 |  |  |  |  |  | 🗸 | 🗸 | 🗸 | C/D | X |
| Karupaiah et al. (2013) |  |  |  |  |  | X | 🗸 | 🗸 | 🗸 | 🗸 |  |  |  |  |  |  |  |  |  |  |
| Kelly et al. (2015) | 🗸 | 🗸 | C/D | 🗸 | 🗸 | 🗸 | 🗸 | 🗸 | 🗸 | 🗸 |  |  |  |  |  | 🗸 | X | 🗸 | 🗸 | 🗸 |
| Kelly et al. (2014) |  |  |  |  |  | X | 🗸 | X | 🗸 | 🗸 |  |  |  |  |  |  |  |  |  |  |
| Lim et al. (2017) |  |  |  |  |  | 🗸 | X | 🗸 | 🗸 | 🗸 |  |  |  |  |  |  |  |  |  |  |
| Lim et al. (2020) | 🗸 | 🗸 | 🗸 | 🗸 | 🗸 | 🗸 | 🗸 | 🗸 | 🗸 | 🗸 |  |  |  |  |  | 🗸 | 🗸 | 🗸 | 🗸 | 🗸 |
| Lipoeto et al. (2013) | 🗸 | C/D | C/D | 🗸 | X | X | C/D | C/D | C/D | C/D |  |  |  |  |  | X | X | 🗸 | X | X |
| Moxley et al. (2011) |  |  |  |  |  | X | X | 🗸 | 🗸 | 🗸 |  |  |  |  |  |  |  |  |  |  |
| Naidoo et al. (2017) | 🗸 | X | 🗸 | 🗸 | 🗸 | 🗸 | 🗸 | 🗸 | 🗸 | 🗸 |  |  |  |  |  | 🗸 | 🗸 | 🗸 | X | 🗸 |
| Neo & Brownlee, (2017) | 🗸 | 🗸 | 🗸 | 🗸 | 🗸 |  |  |  |  |  |  |  |  |  |  |  |  |  |  |  |
| Ng et al. (2015) | 🗸 | C/D | 🗸 | 🗸 | X |  |  |  |  |  |  |  |  |  |  |  |  |  |  |  |
| Ong & Kim, (2017) | 🗸 | 🗸 | C/D | C/D | 🗸 |  |  |  |  |  | 🗸 | C/D | 🗸 | C/D | 🗸 | 🗸 | 🗸 | 🗸 | 🗸 | X |
| Pawera et al. (2020) | 🗸 | 🗸 | 🗸 | 🗸 | 🗸 |  |  |  |  |  | 🗸 | 🗸 | 🗸 | C/D | 🗸 | X | X | 🗸 | 🗸 | 🗸 |
| Pei et al. (2018) |  |  |  |  |  | 🗸 | 🗸 | 🗸 | 🗸 | 🗸 |  |  |  |  |  |  |  |  |  |  |
| Rammohan et al. (2019) |  |  |  |  |  | 🗸 | 🗸 | 🗸 | 🗸 | 🗸 |  |  |  |  |  |  |  |  |  |  |
| Reyes-García et al. (2019) | 🗸 | 🗸 | C/D | 🗸 | 🗸 |  |  |  |  |  | 🗸 | 🗸 | 🗸 | 🗸 | 🗸 | 🗸 | 🗸 | 🗸 | 🗸 | 🗸 |
| Sang-ngoen et al. (2019) |  |  |  |  |  | X | 🗸 | 🗸 | X | 🗸 |  |  |  |  |  |  |  |  |  |  |
| Sang-ngoen et al. (2020) |  |  |  |  |  | X | 🗸 | 🗸 | X | 🗸 |  |  |  |  |  |  |  |  |  |  |
| Schram et al. (2015) |  |  |  |  |  | 🗸 | 🗸 | 🗸 | 🗸 | 🗸 |  |  |  |  |  |  |  |  |  |  |
| Sufyan et al. (2019) | 🗸 | X | 🗸 | 🗸 | 🗸 |  |  |  |  |  |  |  |  |  |  |  |  |  |  |  |
| Supannee, (2020) |  |  |  |  |  | 🗸 | 🗸 | C/D | 🗸 | 🗸 |  |  |  |  |  |  |  |  |  |  |
| Trinh et al. (2020) |  |  |  |  |  |  |  |  |  |  | 🗸 | 🗸 | 🗸 | C/D | 🗸 |  |  |  |  |  |
| Wallace et al. (2014) | 🗸 | 🗸 | C/D | C/D | 🗸 |  |  |  |  |  | 🗸 | X | 🗸 | C/D | 🗸 | X | X | X | C/D | X |
| Wertheim-Heck & Raneri, (2019) | 🗸 | 🗸 | C/D | 🗸 | 🗸 | 🗸 | 🗸 | 🗸 | 🗸 | 🗸 | 🗸 | 🗸 | 🗸 | 🗸 | 🗸 | 🗸 | 🗸 | 🗸 | 🗸 | 🗸 |
| Wertheim-Heck et al. (2014) |  |  |  |  |  | 🗸 | 🗸 | 🗸 | 🗸 | 🗸 | 🗸 | 🗸 | 🗸 | 🗸 | 🗸 |  |  |  |  |  |
| Wertheim-Heck et al. (2019) | 🗸 | 🗸 | C/D | 🗸 | 🗸 | 🗸 | 🗸 | 🗸 | 🗸 | 🗸 | 🗸 | 🗸 | 🗸 | 🗸 | 🗸 | 🗸 | 🗸 | 🗸 | 🗸 | 🗸 |

*Qualitative study questions:* 1.1. Is the qualitative approach appropriate to answer the research question? 1.2. Are the qualitative data collection methods adequate to address the research question? 1.3. Are the findings adequately derived from the data? 1.4. Is the interpretation of results sufficiently substantiated by data? 1.5. Is there coherence between qualitative data sources, collection, analysis and interpretation? *Quantitative non-randomized* *study questions:* 3.1. Are the participants representative of the target population? 3.2. Are measurements appropriate regarding both the outcome and intervention (or exposure)? 3.3. Are there complete outcome data? 3.4. Are the confounders accounted for in the design and analysis? 3.5. During the study period, is the intervention administered (or exposure occurred) as intended? *Quantitative descriptive* *study questions:* 4.1. Is the sampling strategy relevant to address the research question? 4.2. Is the sample representative of the target population? 4.3. Are the measurements appropriate? 4.4. Is the risk of nonresponse bias low (response rate of 60% or above)? 4.5. Is the statistical analysis appropriate to answer the research question? *Mixed-methods questions:* 5.1. Is there an adequate rationale for using a mixed methods design to address the research question? 5.2. Are the different components of the study effectively integrated to answer the research question? 5.3. Are the outputs of the integration of qualitative and quantitative components adequately interpreted? 5.4. Are divergences and inconsistencies between quantitative and qualitative results adequately addressed? 5.5. Do the different components of the study adhere to the quality criteria of each tradition of the methods involved? (Hong et al., 2018) Abbreviations: C/D, cannot determine; X, no; 🗸, yes.

## Table S4: Reference sources used in Figure 5 of manuscript

| Food environment dimension | Summary points | Data Sources |
| --- | --- | --- |
| Availability | - Increase in overall food and retailer availability across region - No significant relationship found between processed food availability and either shopping patterns or diet quality - Perceived food availability dependent on population being studied | Baker and Friel, 2016; Colozza, 2020; Colozza and Avendano, 2019; Downs et al., 2019; Goh et al., 2020; Harris et al., 2020; Schram et al., 2015; Trinh et al., 2020; Wertheim-Heck et al., 2014 |
| Price | - Nutritious, diverse diets are becoming more expensive - High food prices considered a major barrier to consuming a healthier diet, particularly among low-income communities - Substantial differences in food price between food retailers influences food shopping behaviours | Downs et al., 2019; Harris et al., 2020; Kelly et al., 2015; Sufyan et al., 2019; Wallace et al., 2014; Wertheim-Heck et al., 2019 |
| Vendor & Product Properties | - Vendor properties influence food purchasing by catering to different consumer needs - Traditional markets remain the primary food source & preferred vendor type across the region, although use of supermarkets is positively associated with income, urban living, & small households | Anggraini et al., 2016; Baker and Friel, 2016; Kelly et al., 2014, 2015; Sufyan et al., 2019; Trinh et al., 2020; Wertheim-Heck et al., 2014, 2019; Wertheim-Heck and Raneri, 2019 |
| Marketing & regulation | - Trade liberalization linked to increased sales of SSCBs & other ultra-processed foods. It also facilitates market monopolies & the domination of transnational food companies | Baker & Friel, 2016; Schram et al., 2015 |
| Accessibility | - Accessibility is a particular concern among rural & low-income populations due to transportation limitations - Access to natural food environments found to be significantly associated with higher food security & dietary diversity scores - Geographic proximity to food retail outlets not found to significantly influence dietary diversity or food acquisition | Bin Tan and Arcaya, 2020; Bhanbhro et al., 2020; Gan et al., 2020; Kelly et al., 2014; Ong and Kim, 2017; Pawera et al., 2020; Rammohan et al., 2019; Reyes-García et al., 2019; Wertheim-Heck et al., 2019 |
| Affordability | - Affordability considered major barrier to achieving a healthy diet - Household income was significantly & positively correlated with diet quality, food security, & nutrient intake | Bhanbhro et al., 2020; Downs et al., 2019; Gan et al., 2020; Kelly et al., 2015; Pei et al., 2018; Sufyan et al., 2019; Wallace et al., 2014 |
| Convenience | - Time constraints influenced the consumption of ready-to-eat meals & the frequency of eating out - Convenience as a motive for food choice was associated with household income | Abdul Rahman et al., 2013; Asma et al., 2010; Ferzacca et al., 2013; Naidoo et al., 2017; Sufyan et al., 2019 |
| Desirability | - Individual & family food preferences strongly influence household food purchasing decisions - Traditional market shopping thought to build social cohesion - Ethnicity, nutrition knowledge, and level of education were significantly associated with BMI and diet quality | Banwell et al., 2016; Bhanbhro et al., 2020; Chong et al., 2019; Downs et al., 2019; Ferzacca et al., 2013; Fournier et al., 2016; Karupaiah et al., 2013; Kelly et al., 2015; Lim et al., 2020; Moxley et al., 2011; Neo and Brownlee, 2017; Ng et al., 2015; Sufyan et al., 2019; Wertheim-Heck et al., 2014 |
